# Supplementary material for: Association between Expression of Connective Tissue Genes and Prostate Cancer Growth and Progression
Source: Int J Mol Sci. 2023 Apr 19;24(8):7520. doi: 10.3390/ijms24087520 (PMC10139147; doi:10.3390/ijms24087520)
Supplement: Supplementary file 1 [file ijms-24-07520-s001.zip › Supplementary Figure S1.pdf]

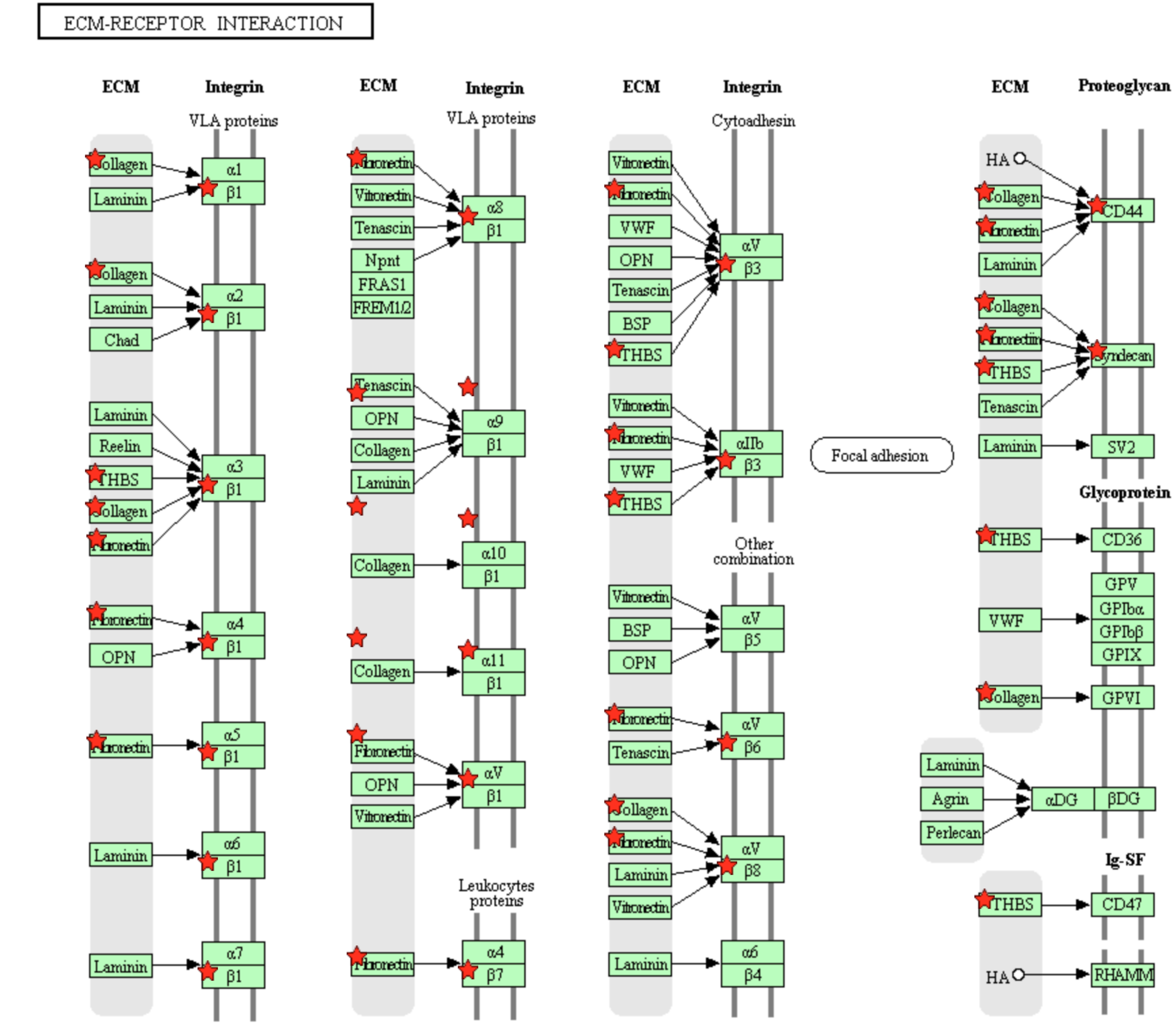

04512 5/15/19
(c) Kanehisa Laboratories

Supplementary Figure S1: Pathway Association analysis: Selection of the connective tissue related genes converge in the ECM-receptor signaling interaction pathway in prostate cancer cells.
