# Supplementary material for: Association between Expression of Connective Tissue Genes and Prostate Cancer Growth and Progression
Source: Int J Mol Sci. 2023 Apr 19;24(8):7520. doi: 10.3390/ijms24087520 (PMC10139147; doi:10.3390/ijms24087520)
Supplement: Supplementary file 1 [file ijms-24-07520-s001.zip › Supplementary Figure S2.pdf]

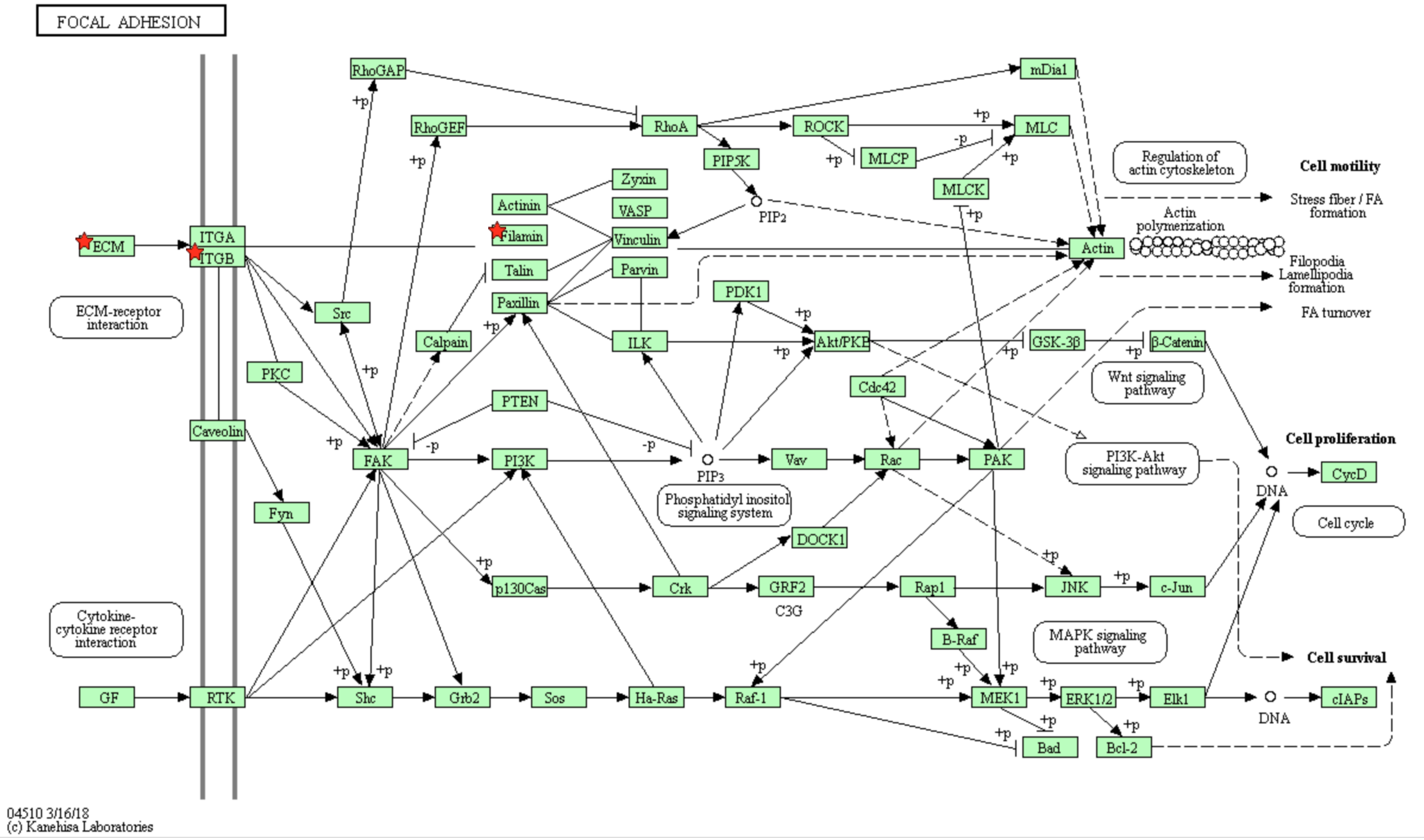
FOCAL ADHESION
ECM
ITGA
ITGB
ECM-receptor interaction
Cytokine-cytokine receptor interaction
GF
RTK
PKC
Caveolin
Fyn
Src
RhoGAP
RhoGEF
FAK
Calpain
Actinin
Filamin
Talin
Paxillin
Zyxin
VASP
Vinculin
Parvin
ILK
PTEN
PI3K
p130Cas
Shc
Grb2
Sos
Ha-Ras
Raf-1
Crk
DOCK1
GRF2
C3G
RhoA
PIP5K
PIP2
PIP3
Phosphatidyl inositol signaling system
PDK1
Akt/PKB
Vav
Rac
Cdc42
PAK
ROCK
MLCP
MLC
MLCK
mDia1
Rap1
B-Raf
MEK1
ERK1/2
Elk1
JNK
c-Jun
Bad
Bcl-2
GSK-3β
β-Catenin
Actin
Regulation of actin cytoskeleton
Actin polymerization
Wnt signaling pathway
PI3K-Akt signaling pathway
MAPK signaling pathway
Cell cycle
Cell motility
Stress fiber / FA formation
Filopodia Lamellipodia formation
FA turnover
Cell proliferation
DNA
CycD
Cell survival
cIAPs
+p
-p
04510 3/16/18
(c) Kanehisa Laboratories

Supplementary Figure S2: Pathway association analysis revealed activation of the focal adhesion pathway, directed by the selected genes regulating (functionally associated with the connective tissue).
